# Comprehensive molecular analysis based on somatic copy number alterations in intramucosal colorectal neoplasias and early invasive colorectal cancers

**DOI:** 10.18632/oncotarget.25112

**Published:** 2018-05-01

**Authors:** Tamotsu Sugai, Makoto Eizuka, Wataru Habano, Yasuko Fujita, Ayaka Sato, Ryo Sugimoto, Kouki Otsuka, Eiichiro Yamamoto, Takayuki Matsumoto, Hiromu Suzuki

**Affiliations:** ^1^ Department of Molecular Diagnostic Pathology, School of Medicine, Iwate Medical University, Morioka, Japan; ^2^ Department of Pharmacodynamics and Molecular Genetics, School of Pharmacy, Iwate Medical University, Morioka, Japan; ^3^ Department of Surgery, Iwate Medical University, School of Medicine, Iwate Medical University, Morioka, Japan; ^4^ Department of Molecular Biology, Sapporo Medical University, School of Medicine, Cyuuouku, Sapporo, Japan; ^5^ Division of Gastroenterology, Department of Internal Medicine, School of Medicine, Iwate Medical University, Morioka, Japan

**Keywords:** colorectal cancer, comprehensive genomic analysis, colorectal adenoma, copy number alteration, microsatellite instability

## Abstract

It is unclear whether somatic copy number alterations (SCNAs) contribute to the development of colorectal cancer (CRC). Here, we aimed to identify the molecular profiles of early colorectal carcinogenesis based on SCNAs and determine the associations of other molecular abnormalities for the detection of neoplasia in both intramucosal neoplasia (IMN) and invasive CRC with invasion into the muscular layer without metastasis (early invasive CRC). A single nucleotide polymorphism array was used to examine 100 colorectal IMNs (low-grade adenoma [LGA], 40; high-grade adenoma [HGA], 25; intramucosal adenocarcinoma [IMA], 35) and early invasive CRC (20 tumors). In addition, genetic mutations (*KRAS*, *BRAF*), TP53 overexpression, microsatellite instability (MSI), and DNA methylation (low, intermediate, high) were examined. Hierarchical clustering analysis based on the SCNA pattern was carried out to identify molecular profiles in IMNs and early invasive CRC. Colorectal tumors were classified into three subgroups based on SCNA patterns. Subgroup 1 was characterized by multiple SCNAs, subgroup 3 was closely associated with infrequent SCNAs, and subgroup 2 was an intermediate subgroup in SCNA pattern between subgroups 1 and 3. Although mutations in *KRAS* were commonly found in all three subgroups, overexpression of TP53 was observed primarily in subgroup 1 and 2. DNA methylation showed a low/intermediate type. Finally, no MSI was detected. Each subgroup was correlated with histology (subgroup 1, early invasive CRC; subgroup 2, LGA; subgroups 2 and 3, HGA and IMA). Considerable SCNAs may be required for acquisition of invasive ability in CRC. Our results provide novel insights into early CRC.

## INTRODUCTION

Colorectal cancer (CRC) is the third most common form of cancer and the second leading cause of cancer-related death worldwide [1]. Most sporadic CRCs arise through the adenoma-carcinoma sequence [2, 3]. A genetic model for the adenoma-carcinoma sequence has been proposed in which the sequential accumulation of mutations in specific genes, including *APC*, *KRAS*, and *TP53*, drives the transition from healthy colonic epithelia through increasingly dysplastic adenoma to colorectal cancer [2, 3]. The identification of early molecular alterations in early colorectal lesions (colorectal adenoma [low grade and high grade], intramucosal cancer, and CRC with early invasion) is important. To improve the diagnosis and treatment outcomes in patients with CRC, it will be necessary to elucidate the molecular alterations associated with early-stage colorectal lesions.

Recent studies have shown that there are two major molecular alterations in cancers, i.e., chromosomal instability (CIN or microsatellite stable [MSS]) and microsatellite instability (MSI; MIN) [3, 4]. The majority of sporadic colon cancers (85%) exhibit chromosomal instability (CIN), which represents the end result of a number of processes, including alterations in mitotic checkpoint genes that may induce somatic copy number alterations (SCNAs) [5, 6]. In contrast, MIN-type CRC shows the presence of high-level microsatellite instability (MSI-H) and the loss of MLH1/PMS2 expression [7]. Furthermore, DNA methylation levels are high or intermediate/low in MIN and CIN CRCs, respectively [3, 7]. Finally, whereas *BRAF* mutations are common in MIN-type CRC, *TP53* mutations are closely associated with CIN (or MSS)-type CRC [3, 5, 7]. Although recent evidence suggests that there may be overlap between the two types of CRC, it is believed that CIN (or MSS) and MIN (or MSI) types are mutually exclusive [3, 4, 8].

SCNAs in the tumor cell genome are a common molecular mechanism of CIN that contributes to cancer development. SCNAs are frequently found in not only CRC but also other gastrointestinal cancers. Although genomically altered regions are very common in human cancers, it is often difficult to identify true cancer-related genes in such amplicons because of the complex network of genes affected. However, recent studies have shown that SCNAs are indicators of chromosomal destruction and play a major role in the development of CRC [9–12].

The incidence and mortality rate of CRC can be reduced by early detection and removal of treatable neoplasia; however, there is a lack of useful markers specific for both established invasive cancer and precancerous lesions [13]. Molecular stratification, combined with other alterations that are related to tumor evolution, may be suitable for evaluation of early colorectal carcinogenesis [13]. Our previous study has shown that SCNAs are progressively associated with the development and progression of premalignant lesions to early invasive CRC (invasion into the muscular layer without metastasis) [14, 15].

Based on this background, the aim of the present study was to identify the molecular profiles of colorectal tumors based on SCNAs and the associations of SCNAs with other molecular alterations related to the development of cancer in colorectal tumors.

## RESULTS

In the present study, hierarchical clustering analysis based on the CNA pattern, including gains, LOHs, and copy-neutral LOHs, was carried out to examine differences in genetic alterations in samples from patients with colorectal IMNs and CRCs that may have invaded into the muscular layer.

**Figure 1:**
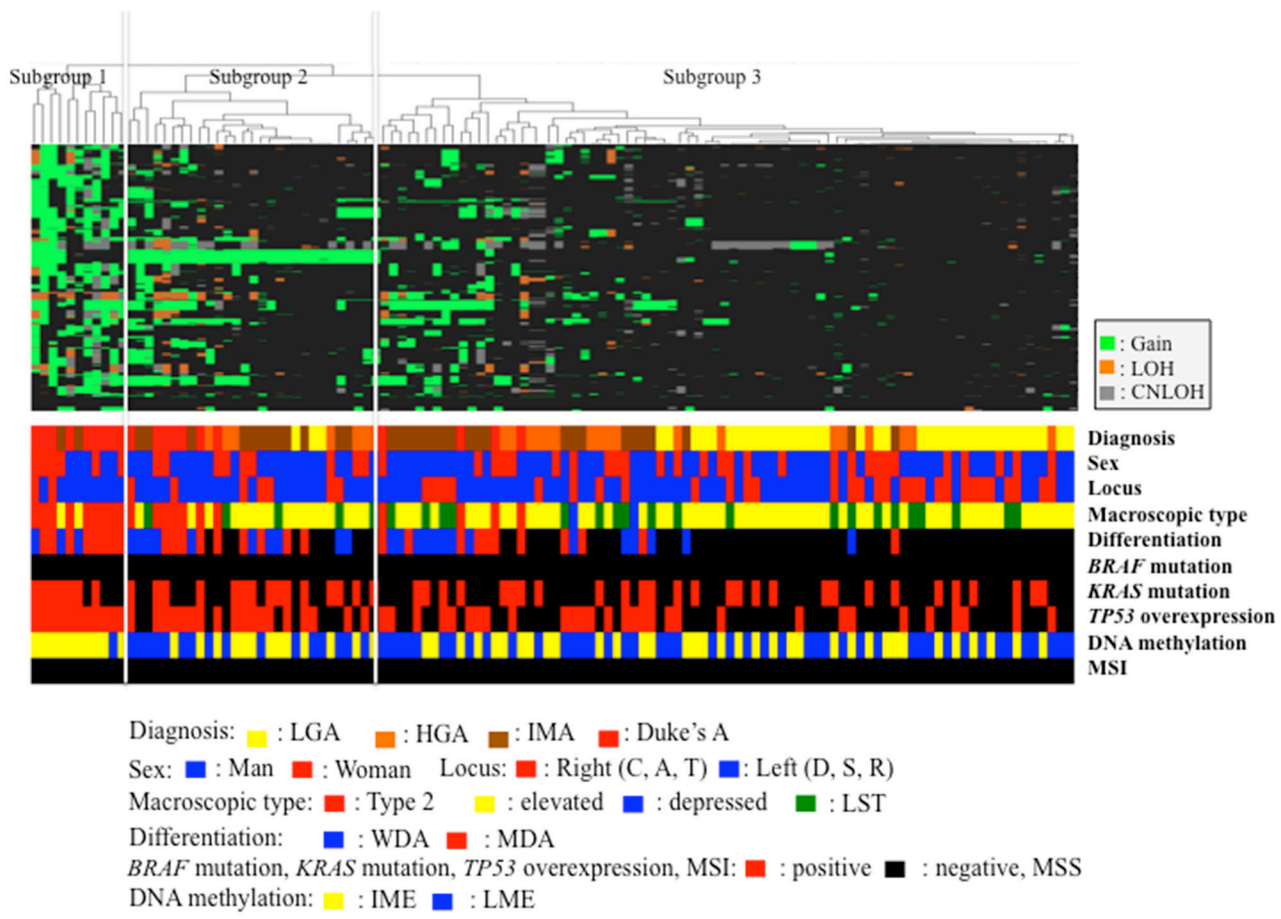
Hierarchical cluster analysis based on somatic copy number alterations in 120 colorectal tumors

Three distinct subgroups were categorized, as shown in Figure 1. The vertical line shows SCNAs, and the horizontal lines denote “relatedness” between samples and CNAs at the chromosomal loci. The colorectal tumors examined in this study were categorized into 3 distinct patterns in the cluster analysis.

The clinical findings in each subgroup categorized based on CNAs are listed in Table 1. The median size of the colorectal tumors examined in this study was significantly higher in tumors in subgroup 1 than in tumors in subgroups 2 or 3. The frequency of LGA was significantly higher in tumors in subgroup 3 (37/80, 46.3%) than in subgroups 1 (0/11; *p* < 0.001) or 2 (3/29, 10.3%; *p* < 0.01). In addition, significant differences in the frequencies of LGA between subgroups 1 and 2 were also observed (*p* < 0.01). However, there were no significant differences in the frequencies of IMA between the three subgroups. Next, there was a significant difference in the frequency of early invasive CRC between subgroups 1 (9/11, 81.8%) and 2 (7/29, 24.1%; *p* < 0.01) or 3 (4/80, 5%; *p* < 0.00). Moreover, a significant difference in the frequency of early invasive CRC was observed between subgroups 1 and 3 (*p* < 0.001). Finally, there were no differences in the frequencies of HGA between subgroups 2 (7/29, 24.1%) and 3 (18/80, 22.5%).

**Table 1:** Clinicopathological findings in subgroups 1, 2, and 3

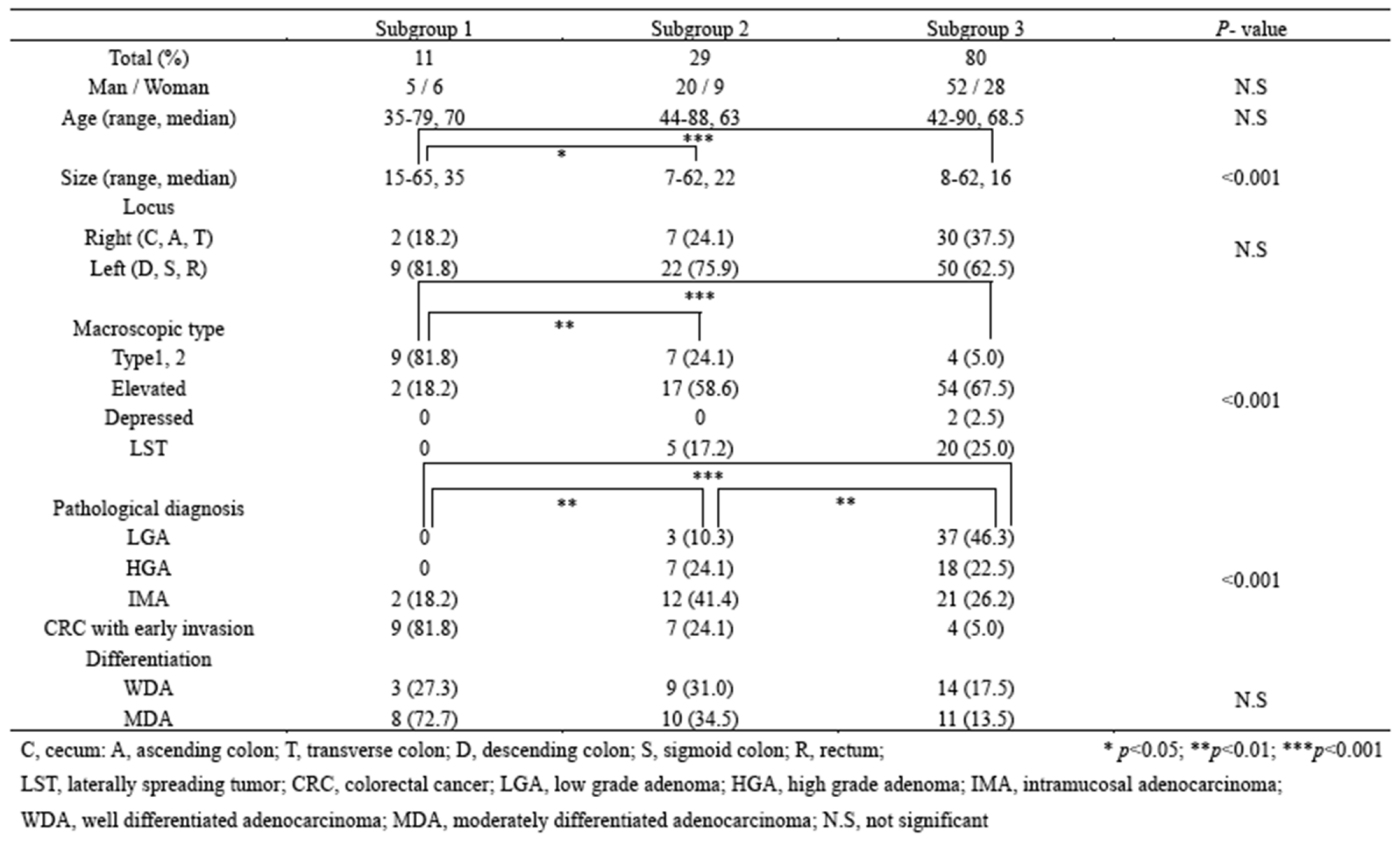

### CNAs in subgroups 1, 2, and 3

**Figure 2:**
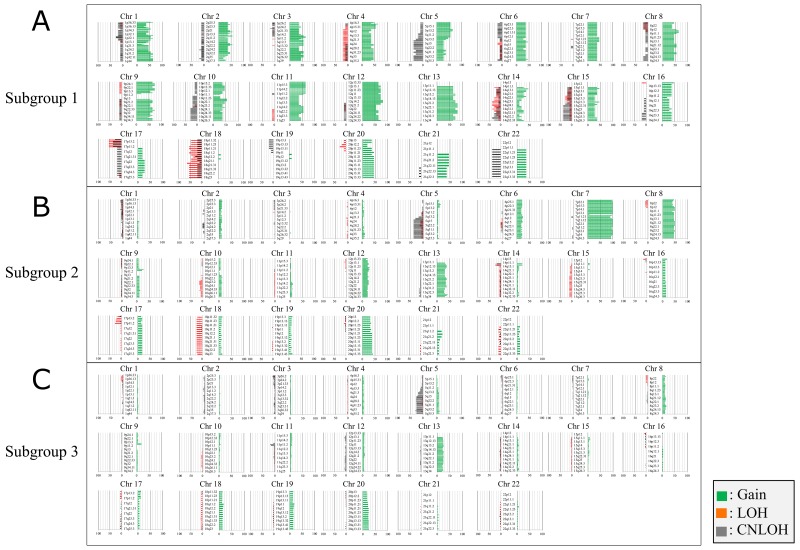
Ideogram of copy number alterations in three subgroups categorized based on somatic copy number alteration patterns in colorectal tumors (intramucosal neoplasia and colorectal cancer with early invasion) Chromosomes are ordered from 1 to 22. The colored horizontal lines represent the frequencies of gains, LOHs, and CNLOHs. Lines on the left indicate losses (red, copy neutral LOH; gray, LOH), and those on the right (green) indicate gains. **(A)** Ideogram of copy number alterations in subgroup 1. **(B)** Ideogram of copy number alterations in subgroup 2. **(C)** Ideogram of copy number alterations in subgroup 3.

The CNAs of all chromosomes according to each subgroup are shown in Figure 2A–2C. The mean total number of chromosomal aberrations per patient was 447, with an average of 327 gains (range: 216–511), 41 LOHs (range: 0–148), and 79 copy-neutral LOHs (range: 0–232) in subgroup 1. In subgroup 3, the mean total number of chromosomal aberrations per patient was 72, with an average of 43 gains (range: 0–199), 8 LOHs (range: 0–138), and 21 copy-neutral LOHs (range: 0–151). Finally, in subgroup 2, the mean total number of chromosomal aberrations per patient was 174, with an average of 130 gains (range: 40–347), 22 LOHs (rang: 0–161), and 22 copy-neutral LOHs (range: 0–86). There were signicant differences in the total numbers of CNAs between subgroups 1 and 2 or 3 (*p* < 0.01). Moreover, significant differences were observed in the average numbers of CN gains between subgroups 1 and 2 or 3 (*p* < 0.01). Although LOH was common betwen the three subgroups, there were significant differences in the average numbers of CN LOHs between subgroups 1 and 3 (*p* < 0.05).

Regions of gains detected in more than 30% of cases were located at 13q11-q34, 12p11.1-p13.33, 12q11-q24.33, 9p13.1-p24,3, 9q21.1-q34.3, 1p11.2-p36.33, 1q21.3-q44, 3p11.1-p26.3, 3q11.1-q29, 4p12-p16.3, 4q13.1-q35.2, 5p11-p15.33, 5q11.1-q13.3, 8p11.1-p23.3, 8q11.1-q24.3, 11p11.2-p15.5, 11q11-q25, 2p11.2-p25.3, 2q11.2-q33.3, 10p11.21-p15.3, 10q11.21-q26.3, 19p12-13.3, 19q11-q13.43, 20p11.1-p11.23, 20q11.21-q13.33, 7p11.2-p22.3, and 7q11.1-q36.3 in subgroup 1 and at 7p11.2-p22.3, 7q11.1-q36.3, 8p11.1-p23.3, 8q11.1-q24.3, 13q11-q31.3, 13q33.1-q34, 20p11.1-p13, and 20q11.21-q13.33 (in decreasing order of frequency) in subgroup 2. No regions of gains were detected in more than 30% of cases in subgroup 3. Although LOHs detected in more than 30% of cases were found at 18p11.21-p11.32, 18q11.1-q23, 17p11.2-p13.3, 14q23.1-q24.1, and 14q31 in subgroup 1, no LOH (more than 30% of cases) was detected in subgroups 2 and 3. Moreover, copy-neutral LOHs (more than 30% of cases) were found at 5q14.1-23.1, 5q31.1-q31.2, 5q33.1-q35.3, 14q32.31-q32.32, 15q22.31-q26.3, 17p11.2-p13.2, and 22q11.1-q13.3 in subgroup 1 and at 5q14.1-q35.3 and 5q14 in subgroups 2 and 3, respectively. These results are summarized in Table 2.

**Table 2:** Frequent CNA regions in each subgroup

Chromosomal regions	Subgroup 1 n=11 (%)
**Gain**	
13q11-q34	7-9 (63.6-81.1)
12p11.1-p13.33, 12q11-q24.33	6-9 (54.5-81.8)
9p13.1-p24.3, 9q21.1-q34.3	5-8 (54.5-72.7)
1p11.2-p36.33, 1q21.3-q44	4-7 (36.4-63.6)
3p11.1-p26.3, 3q11.1-q29	4-7 (36.4-63.6)
4p12-p16.3, 4q13.1-q35.2	4-7 (36.4-63.6)
5p11-p15.33, 5q11.1-q13.3	4-7 (36.4-63.6)
8p11.1-p23.3, 8q11.1-q24.3	4-7 (36.4-63.6)
11p11.2-p15.5, 11q11-q25	4-7 (36.4-63.6)
2p11.2-p25.3, 2q11.2-q33.3	4-6 (36.4-54.5)
10p11.21-p15.3, 10q11.21-q26.3	4-6 (36.4-54.5)
19p12-13.3, 19q11-q13.43	4-6 (36.4-54.5)
20p11.1-p11.23, 20q11.21-q13.33	4-5 (36.4-45.5)
7p11.2-p22.3, 7q11.1-q36.3	4-5 (36.4-45.5)
**CNLOH**	
5q14.1-23.1, 5q31.1-q31.2 5q33.1-q35.3	4 (36.4) 4 (36.4)
14q32.31-q32.32	4 (36.4)
15q22.31-q26.3	4 (36.4)
17p11.2-p13.2	4 (36.4)
22q11.1-q13.3	4 (36.4)
**LOH**	
18p11.21-p11.32, 18q11.1-q23	4-7 (36.4-63.6)
17p11.2-p13.3	6 (54.5)
14q23.1-q24.1, 14q31.1	4 (36.4)

Chromosomal regions	Subgroup 2 n=29 (%)
**Gain**	
7p11.2-p22.3, 7q11.1-q36.3	26-29 (89.7-100)
8p11.1-p23.3, 8q11.1-q24.3	11-14 (37.9-48.3)
13q11-q31.3, 13q33.1-q34	9-12 (31.0-41.4)
20p11.1-p13, 20q11.21-q13.33	9-12 (31.0-41.4)
**CNLOH**	
5q14.1-q35.3	9-11 (31.0-37.9)
**LOH**	
none	

Chromosomal regions	Subgroup 3 n=80 (%)
**Gain**	
none	
**CNLOH**	
5q34	24 (30.0)
**LOH**	
none	

### Differences in CNAs between subgroups

Next, we examined differences in CNAs between the three subgroups. Regions of gain detected in more than 30% of cases were selected for comparison of each group.

Significant differences in gains between subgroups 1 and 2 were found at 1p, 1q, 2p, 3p, 3p, 4p, 4q, 5p, 5q, 7p, 7q, 9p, 9q, 10q, 11p, 11q, 12p, 12q, 13q, and 15q (subgroup 1 > 2; Supplementary Table 1). Significant differences in the frequencies of copy-neutral LOH between subgroups 1 and 2 were found at 15q22.31-q26.3 and 17p13.1-p13.2 (subgroup 2 < 1). However, no differences in the frequencies of LOH were found between the two subgroups.

Significant differences in the frequencies of copy number gains were observed between subgroups 2 and 3 at 6p, 6q, 7p, 7q, 8p, 8q, 12p, and 12q (Supplementary Table 2). Although no significant differences in copy-neutral LOH were observed between subgroups 2 and 3, significant differences in LOH at 14q11.2, 14q12, 17p11.2, and 18q11.1-q12 were found between subgroups 2 and 3.

Finally, we examined significant differences in the frequencies of CNAs between subgroups 1 and 3. Significant differences in copy number gains between subgroups 1 and 3 were observed at 1p, 1q.3q, 2p, 2q, 3p, 3q, 4p, 4q2, 5q, 5p, 6p, 6q, 7p, 7q, 8p, 8q, 9p, 9q, 10p, 10q, 11p, 11q, 12p, 12q, 13q, 14q, 15q, 16p, 16q, 19p, 19q, 21q, and 22q (subgroup 1 > 3; Supplementary Table 3). There were significant differences in the frequencies of copy-neutral LOH at 15q, 22q, 17p, 10q, 1p and 8p between subgroups 1 and 3. In addition, significant differences in the frequencies of copy-neutral LOH at 17p, 18p, 18q, 14q, 20p, 6q, 14q, and 15q were found between subgroups 1 and 3.

### Association of the lengths of CNAs on the genome-wide scale in subgroups 1, 2, and 3

**Figure 3:**
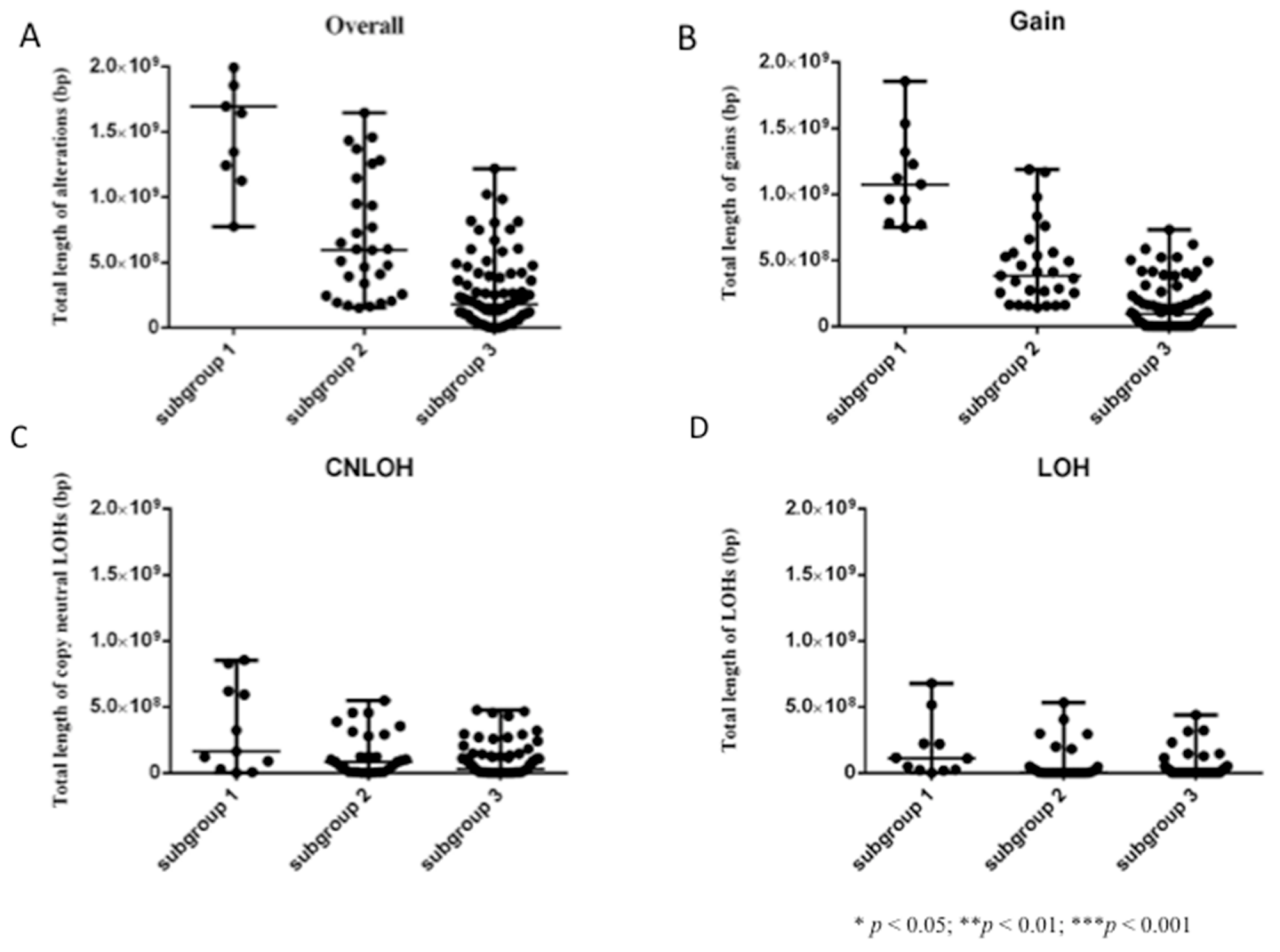
Comparison of the total lengths of SCNAs, SCNA gains, SCNA copy-neutral LOHs, and SCNA LOHs in 120 colorectal tumors that were categorized based on SCNAs **(A)** Comparison of the total lengths of overall SCNAs in the three subgroups. **(B)** Comparison of the total lengths of gains of SCNAs in the three subgroups. **(C)** Comparison of the total lengths of copy-neutral LOHs of SCNAs in the three subgroups. **(D)** Comparison of the total lengths of LOHs of SCNAs in the three subgroups.

Overall, the total lengths of CNAs were longer in subgroup 1 than in subgroups 2 or 3 (Figure 3; *p* < 0.0001). There were significant differences in the lengths of CNAs between subgroups 2 and 3. We analyzed genomic losses (LOH and copy-neutral LOH) and gains separately. The total lengths of CNA gains were significantly longer in subgroup 1 than in subgroups 2 or 3 (Figure 3; *p* < 0.0001). In addition, there were significant differences in the total lengths of CNA gains between subgroups 2 and 3. In contrast, the total lengths of copy-neutral LOH were significantly longer in subgroup 1 than in subgroup 3 (Figure 3; *p* = 0.0045 and *p* < 0.0001, respectively). Furthermore, significant differences in the total lengths of LOHs were found between subgroups 1 and 3 (Figure 3). Finally, significant differences in the total lengths of LOHs were observed between subgroups 1 and 2.

### Differences in MSI, mutations in cancer-related genes, and methylation statuses between subgroups 1, 2, and 3

Tumors with MSI were not found in the present study. Thus, we next examined mutations in *KRAS* and *BRAF* and overexpression of *TP53* in subgroups 1, 2, and 3. No *BRAF* mutations were detected in the colorectal tumors (IMN and CRC) examined in this study. Although mutations in the *KRAS* gene were frequently found in subgroups 1 (7/11, 63.6%) and 2 (15/29, 51.7%), compared with that in subgroup 3 (28/80, 35%), the association did not reach significance (*p* = 0.09). In addition, the frequency of *TP53* overexpression was significantly higher in subgroups 1 and 2 than in subgroup 3 (*p* < 0.01; *p* < 0.05). Finally, we analyzed methylation statuses in subgroups 1, 2, and 3 and found that there were differences in the frequencies of HME or LME methylation statuses between subgroups 1 and 2 or 3. These results are summarized in Table 3.

**Table 3:** Molecular findings in subgroup 1, 2 and 3

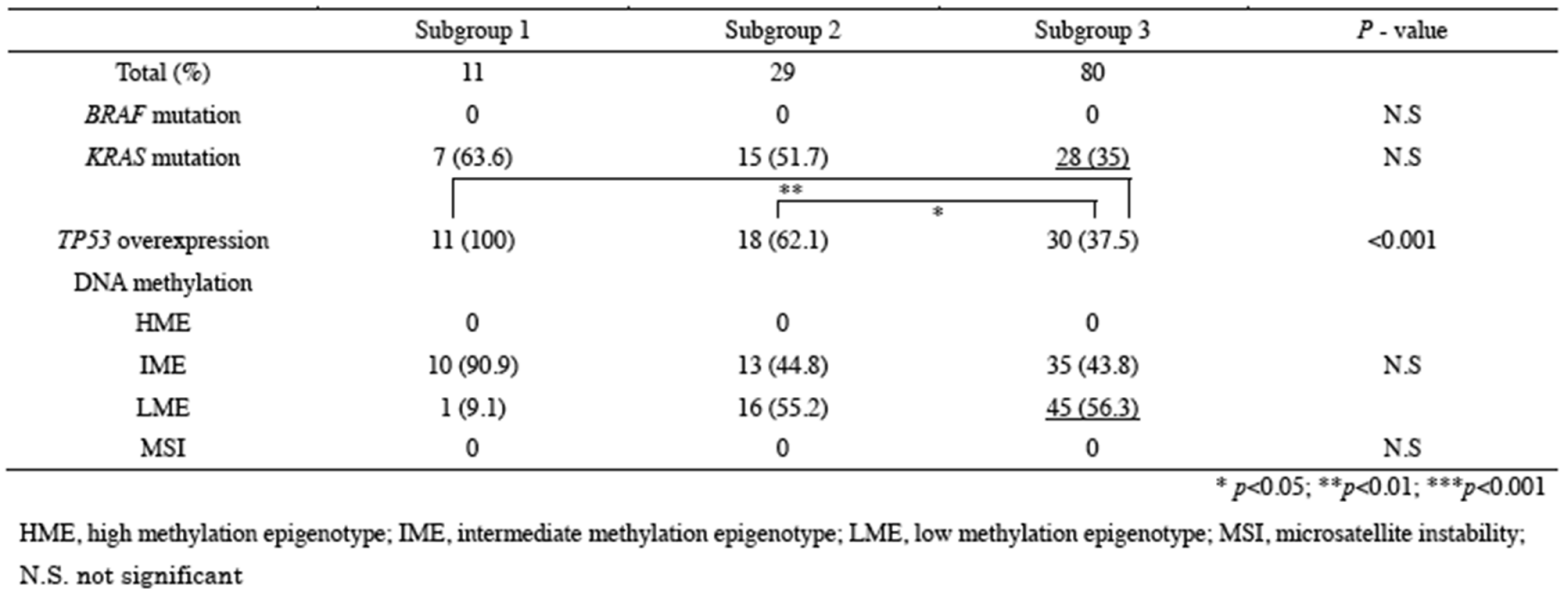

## DISCUSSION

In our previous studies, we have shown that SCNAs, which are changes in genomic DNA that result in aggressive characteristics in tumor cells, contribute significantly to cancer progression [15, 16]. In addition, Eizuka et al. indicated that there were significant differences based on SCNA patterns among LGA, HGA, and IMA [15]. In the present study, we performed hierarchical clustering analysis based on SCNAs using high-throughput genome-wide analysis to identify molecular characteristics during early colorectal tumorigenesis. Consequently, we identified three distinct subgroups based on the frequency of SCNAs. The current study provides an overview of genomic alterations present in MNs and early invasive CRCs. These results may improve our understanding of the role of SCNAs in early colorectal carcinogenesis.

Previous studies have shown that extensive genome-wide chromosomal alterations, indicative of CIN, are found in the vast majority of CRCs [9–13]. However, the role of early colorectal carcinogenesis, including IMNs and early invasive cancer, has been poorly understood [15, 17]. In the present study, tumors in subgroup 1 exhibited the CIN type, which is characterized by multiple SCNAs, and were primarily composed of early invasive CRCs. This finding indicated that multiple SCNAs may trigger CIN, resulting in invasion beyond the mucosal layer. Accordingly, we suggest that considerable accumulation of SCNAs may be required for early colorectal invasion. These data were supported by the finding that aneuploidy as a hallmark of CIN occurs at an early stage in colorectal carcinogenesis [18]. Finally, this observation showed that genetic instability dramatically increased with the accumulation of SCNAs during early progression in CRC.

Chromosomal alterations characterized by SCNAs are infrequently detected in colorectal adenomas [15]. Consistent with this, in the present study, LGA was closely associated with subgroup 3 tumors, characterized by a low frequency of SCNAs [15]. This finding supports that most LGA is genetically stable and exhibits an indolent course during tumor evolution. In contrast, the CIN type, in which SCNAs are frequently found, may be present in HGA, as supported by the finding that there is no significant difference in the accumulation of SCNAs between HGA and IMA [15]. In the present study, HGAs and IMAs were commonly assigned into subgroups 2 and 3, which were defined as having intermediate and low frequencies of accumulation of SCNAs, respectively. As described above, further accumulation of SCNAs may be required to acquire the ability for progression beyond mucosal invasion. To the best of our knowledge, there are very few studies addressing molecular patterns of SCNAs in IMNs. Based on our findings, we suggest that a common molecular mechanism underlies both HGA and IMA.

There is a major discrepancy in the histological diagnosis of colorectal IMN between Western and Japanese pathologists [19]. This difference results in histological assessment of intramucosal stromal invasion of the tumor cell [19, 20]. New histological findings for stromal invasion of gastrointestinal IMNs have been published by the World Health Organization (WHO) [20]. This histological reference for assessment of stromal invasion does not to require the desmoplastic reaction or removal of isolated tumor cells from tumor glands, which are typically considered mandatory histological findings for stromal invasion [20, 21]. The present findings suggested that HGA may share accumulation of SCNAs with IMA that invades into the mucosal interstitium. Accordingly, we suggest that malignant potential defined by accumulation of SCNAs may have already been acquired at the time of progression to HGA.

Although genetic pathways are thought to be closely associated with specific genetic alterations [2, 3], subgroup 2 was characterized by gains in 7p11.2-p22.3 and 7q11.1-q36.3. Subgroup 2 is an important molecular subtype for evaluating early colorectal carcinogenesis, given that subgroup 2 may be characterized by molecular alterations in CRC with malignant potential. Although there are many genes located at 7p11.2-p22.3 and 7q11.1-q36.3, three candidate genes were selected in previous literature: *RAS-related C3 botulinus toxin substrate 1* (*RAC1* located at 7p22) [22], *mitotic arrest deficient-like 1* (*MAD1L1* located at 7p22) [22, 23], and *Huntingtin-interacting protein 1 (HIP1* located at 7q11) [24]. Overexpression of Rac1 leads to increased growth of human CRC cells, whereas downregulation of Rac1 expression by siRNA interferes with cancer progression [22]. These findings suggest that Rac1 plays an important role in signal transduction pathways relevant to human CRC progression [22]. *MAD1L1*, whose dysfunction is associated with chromosomal instability, plays a pathogenic role in some human cancers and may be involved in cancer progression and metastasis [23, 24]. Huntingtin-interacting protein 1 (HIP1) is a cofactor in clathrin-mediated vesicle trafficking [25]. Although it was first implicated in cancer biology as part of a chromosomal translocation in leukemia, HIP1 represents a putative prognostic factor in human cancers, including prostate cancer and CRC [25]. Thus, gains at 7p11.2-p22.3 and 7q11.1-q36.3 play a major role in a subset of IMNs and early invasive CRCs.

Early CRC arises through DNA methylation [26]. Although DNA methylation is important in the serrated pathway [7], DNA methylation may also be altered in conventional adenomas, which are precursors of CRC [27, 28]. In the present study, we showed that low to intermediate DNA methylation was commonly found in IMNs. Accordingly, we suggest that aberrant DNA methylation may occur frequently in colorectal IMNs and early invasive CRC.

*TP53* mutations and overexpression play essential roles in colorectal carcinogenesis [18, 29]. It is widely accepted that TP53 overexpression is closely associated with *TP53* mutations in CRCs [18, 29]. In the present study, *TP53* overexpression was more closely associated with subgroups 1 and 2 than with subgroup 3. This finding suggested that *TP53* overexpression may be correlated with accumulation of SCNAs. The principal function of wild-type TP53 protein is to stabilize cellular function by regulating the cell cycle and inhibiting apoptosis [30]. Most SCNAs arise through nonallelic homologous recombination in which unmatched regions are mistakenly recombined during meiosis [9]. This theory may be linked to cellular instability caused by *TP53* alterations (mutation or overexpression) [31]. Thus, overexpression of *TP53* may replace accumulation of SCNAs for identification of chromosomal instability in tumor cells.

In a previous study, we showed that advanced CRC could be stratified into three subgroups, including low-frequency SCNAs and high-frequency SCNAs, the latter of which were further subclassified into two subgroups. Significant differences in the specific alterations of SCNAs between subgroups 2 and 3 were found (gains at 1q23-44, 1p11-36, 10q11-26, 10p11-13, 12q24-24, and 13q33-33 in subgroup 2 and copy-neutral LOH at 12p12-13, 1q24-25, and 10q22 in subgroup 3). The association of the molecular profiles of advanced-stage CRC with that of early-stage colorectal carcinogenesis is very interesting, and identification of such a relationship would provide novel insights into the molecular pathogenesis of CRC.

The Cancer Genome Atlas (TCGA) has been used as a reliable reference of a comprehensive set of molecular analyses in human cancers [17]. Data from TCGA cannot directly compare with that of the present study because the platform for TCGA was different from that of the present study. In the present study, we targeted early colorectal lesions for comprehensive molecular alterations based on SCNAs. Molecular alterations in these lesions have not been fully examined, even in TCGA. We believe that this is the first study to examine molecular alterations based on SCNAs in early colorectal lesions.

In conclusion, we demonstrated that IMNs and early invasive CRCs had a novel molecular profile for the microsatellite stable pathway (or CIN pathway). Our SNP array data showed that IMNs and early invasive CRCs contained varying levels of CIN in the form of SNCAs and could be divided into three subgroups based on SCNAs. This molecular profiling based on SCNAs could provide novel insights for evaluating early colorectal carcinogenesis.

## MATERIALS AND METHODS

### Patients

Tumor samples and normal colonic mucosa were obtained from resected specimens of 100 patients with intramucosal neoplasia (IMN) and 20 patients with CRC that invaded into the muscular layer without metastasis (early invasive CRC). IMN includes low-grade adenoma (LGA), high-grade adenoma (HGA), and intramucosal adenocarcinoma (IMA). IMN was evaluated according to the modified World Health Organization (WHO) 2010 criteria [21]. Briefly, LGA was characterized by a uniform monolayer of columnar cells with basal nuclei showing minimal atypia. In HGA, nuclear atypia was more frequent, with nuclear pleomorphism, nuclear enlargement, and pseudostratification without stromal invasion. In IMA, there was marked cytological atypia and complex architecture with cribriform groups, irregular branching, glandular anastomosis, and budding of neoplastic cells into the lumen, which were considered representative of stromal invasion. Early invasive CRC was defined as tumors that invaded into the muscular layer. Clinicopathological findings were recorded according to the General Rules for Management of the Japanese Colorectal Cancer Association (Table 4) [32].

**Table 4:** Clinicopathological findings of the examined colorectal tumors

	LGA	HGA	IMA	Duke’s A
Total (%)	40	25	35	20
Man / Woman	29 / 11	11 / 14	27 / 8	10 / 10
Age (range, median)	42-82, 67	53-90, 69	48-88, 64	35-88, 63.5
Size (range, median)	8-62, 13.5	7-53, 18	9-41, 16	21-65, 33
Locus				
Right (C, A, T)	17 (42.5)	7 (28.0)	11 (31.4)	4 (20.0)
Left (D, S, R)	23 (57.5)	18 (72.0)	24 (68.6)	16 (80.0)
Macroscopic type				
Type1, 2	0	0	0	20 (100)
Elevated	34 (85.0)	18 (72.0)	21 (60.0)	0
Depressed	0	0	2 (5.7)	0
LST	6 (15.0)	7 (28.0)	12 (34.3)	0
Differentiation				
WDA			24 (68.6)	2 (10.0)
MDA			11 (31.4)	18 (90.0)
Adenoma component				
TA	29 (72.5)	13 (52.0)		
TVA	11 (27.5)	12 (48.0)		

LGA, low grade adenoma; HGA, high grade adenoma; IMA, intramucosal adenocarcinoma; C, cecum; A, ascending colon; T, transverse colon; D, descending colon; S, sigmoid colon; R, rectum; LST, laterally spreading tumor; WDA, well differentiated adenocarcinoma; MDA, moderately differentiated adenocarcinoma; TA, tubular adenoma; TVA, tubulovillous adenoma.

This study was approved by the local ethics committee of Iwate Medical University (approval number HGH28-26), and all patients provided informed consent.

### Crypt isolation method

Fresh tumor and normal tissues were obtained from resected tumor tissues. Normal colonic mucosa was collected from the most distal portion of the colon.

Crypt isolation from the tumor and normal mucosa was performed as previously described [33]. Briefly, fresh tissues were minced with a razor into small pieces and incubated at 37°C for 30 min in calcium- and magnesium-free Hanks’ balanced salt solution (CMF) containing 30 mM ethylenediaminetetraacetic acid (EDTA). The isolated crypts were immediately fixed in 70% ethanol and stored at 4°C until used for DNA extraction. The fixed isolated crypts were observed under a dissecting microscope (SZ60; Olympus, Tokyo, Japan). The CRC samples were collected primarily from the central area of tumor ulceration. Some isolated crypts were routinely processed by histopathological analysis to confirm the histological nature of the isolated glands. Contamination, such as interstitial cells, was not evident in any of our 120 samples.

### DNA extraction

For each patient, DNA was extracted from isolated tumors and normal glands using classical phenol-chloroform extraction.

### Analysis of MSI

The MSI status was determined using a consensus panel of five reference microsatellite markers (BAT25, BAT26, D2S123, D3S546, and D17S250) by a previously described method [34]. When no marker was altered, the tumors were defined as MSS. When only one marker was altered, the tumors were defined as low MSI. When two or more markers were altered, the tumors were defined as high MSI.

### Analysis of KRAS and BRAF mutations

Mutations in *KRAS* (codons 12 and 13) and *BRAF* (V600E) genes were analyzed using a CE-IVD marked PyroMark (Qiagen, Hilden, Germany) according to the manufacturer’s protocols (Therascreen KRAS Pyro Kit Handbook, version 1, July 2011). The primers used in the present study were described previously [35]. The cut-off value for the mutation assay was 15% mutant alleles [35]. Polymerase chain reaction (PCR) products were examined using a PyroMark Q24 instrument (Qiagen) with PyroMark Q24 1.0.6.3 software.

### Immunohistochemistry for TP53 protein

Immediately after excision, specimens were fixed in 10% neutral-buffered formalin, embedded in paraffin wax, cut into 3-μm-thick paraffin sections, and stained with hematoxylin and eosin (HE) for routine light microscopy. For immunohistochemical staining, additional 3-μm-thick sections were cut from paraffin-embedded tissue and placed on poly-l-lysine-coated glass slides. Sections were deparaffinized in xylene and dehydrated. For determination of TP53 alterations, immunostaining was carried out to detect TP53 protein (clone DO7; DAKO, Carpinteria, CA, USA) using the DAKO Envision+ system, consisting of dextran polymers conjugated with horseradish peroxidase (DAKO), as previously described [36]. The specimens were heated by microwaving (H2500 Microwave Processor; Bio-Rad, Hercules, CA, USA) in citrate buffer (pH 6.0) 3 times for 5 min each at 750 W and then reacted with antibodies. Hematoxylin was used as the counterstain.

In the present study, the intensity of TP53 staining was classified into 3 categories: low, intermediate, and strong. Intermediate and strong positivity for TP53 overexpression was considered “positive overexpression”. Immunopositive results in more than 30% of positive tumor cells were regarded as positive, and immunopositive results for 30% or less of tumor cells were regarded as negative, in accordance with previous reports.

### Pyrosequencing for evaluation of methylation

The DNA methylation status was determined by PCR analysis of bisulfite-modified genomic DNA (EpiTect Bisulfite Kit; Qiagen) using pyrosequencing for quantitative methylation analysis (Pyromark Q24; Qiagen NV). The primers used in this study were designed previously [14].

DNA methylation was quantified using 6 specific promoters originally described by Yagi and colleagues [27, 28]. Briefly, after methylation analysis of the first panel of 3 markers (*RUNX3*, *MINT31*, and *LOX*), tumors with hypermethylated epigenomes (HMEs) were identified based on methylation with at least 2 methylated markers. The remaining tumors were examined using a second panel of 3 markers (*NEUROG1*, *ELMO1*, *and THBD*). Tumors with intermediate methylated epigenomes (IMEs) were defined as those with at least 2 methylated markers, whereas tumors not classified as having HMEs or IMEs were designated as showing hypomethylated epigenomes (LMEs); that for the methylation assay was 30% of tumor cells, as previously reported [14].

### CNA analysis

Extracted DNA was adjusted to a concentration of 50 ng/μL. All 120 paired samples were assayed using an Infinium HumanCytoSNP-12v2.1 BeadChip (Illumina, San Diego, CA, USA), which contains 299,140 single nucleotide polymorphism (SNP) loci, according to the Illumina Infinium HD assay protocol. BeadChips were scanned using iScan (Illumina) and analyzed using GenomeStudio software (v.2011.1; Illumina). The log R ratio (LRR) and B allele frequency (BAF) for each sample were exported from normalized Illumina data using GenomeStudio. Data analysis was performed using KaryoStudio 1.4.3 (CNV Plugin v3.0.7.0; Illumina) with default parameters. Copy number variations (CNVs) were classified as described below. In the classification of chromosome CNVs by CNV partition algorithms, LRR 0 indicated a normal diploid region, LRR greater than 0 indicated a copy number gain, and LRR less than 0 indicated a copy number loss-of-heterozygosity (LOH). BAF values ranged from 0 to 1; homozygous SNPs had BAFs near 0 (A-allele) or 1 (B-allele), and heterozygous diploid region SNPs had BAFs near 0.5 (AB genotype). Additionally, LRR and BAF data were used to identify regions of hemizygous and copy-neutral LOH.

### Calculation of the lengths of CNAs on a genome-wide scale in CRCs

To quantify CNAs on a genome-wide level, we calculated the total lengths of CNAs (losses + gains), total lengths of CNA gains, total lengths of CNA LOHs, and total lengths of CNV-copy neutral LOHs identified by the SNP-array analysis, as previously described [37]. We therefore used the total CNV length as an index representing the degree of chromosomal alterations and assessed the relationship between CNA length (total CNA, CNV gain, CNA LOH, and CNA copy-neutral LOH) and each subgroup, as defined by the specific genetic category in the cluster analysis.

### Statistical analysis

Hierarchical analysis was performed for clustering the samples according to the CNA pattern in order to achieve maximal homogeneity for each group and the highest difference between groups. The clustering algorithm was set to centroid linkage clustering, the standard hierarchical clustering method used in biological analyses. The method was described elsewhere.

Data obtained for histological features, mutations, methylation, and CNA status based on each subgroup were analyzed using chi-square tests with Yates’ corrections with the aid of Stat Mate-III software (Atom, Tokyo, Japan). Differences in age distributions between the 2 groups were analyzed using *Mann-Whitney U* tests (PRISM6; GraphPad software, La Jolla, CA, USA). Differences with *p* values of less than 0.05 were considered significant.

## SUPPLEMENTARY MATERIALS TABLES


