# Supplementary material for: Comprehensive molecular analysis based on somatic copy number alterations in intramucosal colorectal neoplasias and early invasive colorectal cancers
Source: Oncotarget. 2018 May 1;9(33):22895–906. doi: 10.18632/oncotarget.25112 (PMC5955401; doi:10.18632/oncotarget.25112)
Supplement: Supplementary file 1 [file oncotarget-09-22895-s001.pdf]

Comprehensive molecular analysis based on somatic copy number alterations in intramucosal colorectal neoplasias and early invasive colorectal cancers

SUPPLEMENTARY MATERIALS

Supplementary Table 1: Significant differences in the frequencies of CNAs between subgroups 1 and 2

	Subgroup 1 n=11 (%)	Subgroup 2 n=29 (%)	P-value
Gain			
1p11.2-31.2,p32.3-p36.11, p36.32-33	6-7 (54.5-63.6)	1 (3.4)	<0.01
1q25.3-q44	5-7 (45.5-63.6)	0	<0.01
3p11.1-p13,p24.1, p24.3, 3q11.2-q29	5-7 (45.5-63.6)	0	<0.01
4p15.1-p16.1	6-7 (54.5-63.6)	1-2 (3.4-6.9)	<0.01
5p11-p13.3, 5p15.2-p15.33, 5q11.1-q12.3	6-7 (54.5-63.6)	1-2 (3.4-6.9)	<0.01
7p11.2-p22.3, 7q11.23-q36.3	4-5 (36.4-45.5)	28-29 (96.6-100)	<0.01
9p13.3-p24.3	7-8 (63.6-72.7)	2 (6.9)	<0.01
9q21.11-q21.31, q22.31-q31.3, q34.13-34.3	7-8 (63.6-72.7)	3 (10.3)	<0.01
11p11.12-p15.5	6-7 (54.5-63.6)	0-1 (0-3.4)	<0.01
12q14.1-q14.3	9 (81.8)	6-7 (20.7-24.1)	<0.01
1p31.3-p32.2, 1p36.13-p36.31, 1q21.3-q25.2	4-5 (36.4-45.5)	0-1 (0-3.4)	<0.05
2p11.2-12, 2p16.1	6 (54.5)	2 (6.9)	<0.05
3q11.1, 3p14.1-p23, 3p24.2, 3p25.1-p26.3	4-5 (36.4-45.5)	0-1 (0-3.4)	<0.05
4q28.3-q32.2, 4q34.3, 4q35.1	5 (45.5)	1-2 (3.4-6.9)	<0.05
5p14.1-p14.2, 5p15.1, 5q13.1-q13.2	5-6 (45.5-54.5)	1-3 (3.4-10.3)	<0.05
7q11.21-q11.22	5 (45.5)	3 (10.3)	<0.05
9q21.32-q22.2, 9q32, 9q33.2-q34.11	6-7 (54.5-63.6)	3 (10.3)	<0.05
10p12.1-p13, 10q21.1-q21.2	5-6 (45.5-54.5)	2-3 (6.9-10.3)	<0.05
11q14.1-q23.1, 11q24.2-q25	5-6 (45.5-54.5)	2-3 (6.9-10.3)	<0.05
12p11.21-p12.1, 12p13.1, 12q15-21.1	8 (72.7)	6-7 (20.7-24.1)	<0.05
13q31.2-q32.1	9 (81.8)	8-9 (27.6-31.0)	<0.05
15q11.2-q13.2, q14, q21.3, q 22.2, 25.3-q26.3	4-5 (36.4-45.5)	0	<0.05
CNLOH			
15q22.31-q26.3, 17p13.1-p13.2	4 (36.4)	0	<0.05

Supplementary Table 2: Significant differences in the frequencies of CNAs between subgroup 2 and 3

	Subgroup 2 n=29 (%)	Subgroup 3 n=80 (%)	*P*-value
Gain			
7p11.2-p22.3, 7q11.1-q36.3	27-29 (93.1-100)	0-3 (0-3.8)	<0.001
8p11.1-23.3, 8q11.1-q24.23	11-14 (37.9-48.3)	7-12 (8.8-15.0)	<0.01
6p23-p25.3, 6q11.1-12, 6q15, 6q25.1-25.2, 6q26	6-7 (20.7-24.1)	1-2 (1.3-2.5)	<0.01
6p11.1-p11.2, 6p22.1-p22.3, 6q13-q14.1,	5-6 (17.2-20.7)	1-2 (1.3-2.5)	<0.05
6q16.1, 6q21-q22.31, 6q22.33, 6q24.2-24.3	5-6 (17.2-20.7)	1-2 (1.3-2.5)	<0.05
12p11.1, 12p11.22, 12q11-q12, q21.33	7-8 (24.1-27.6)	4-5 (5.0-6.3)	<0.05
CNLOH			
None			
LOH			
14q11.2	7 (24.1)	2 (2.5)	<0.01
14q12	7 (24.1)	3 (3.8)	<0.05
17p11.2	8 (27.6)	5 (6.3)	<0.05
18q11.1-q12.2	7 (24.1)	3-4 (3.8-5.0)	<0.05

Supplementary Table 3: Significant differences in the frequencies of CNAs between subgroups 1 and 3

	Subgroup 1 n=11 (%)	Subgroup 3 n=80 (%)	P- value
Gain			<0.01
1p, 1q21.3q-44	4-7 (36.4-63.6)	0-1 (0-1.3)	<0.01
2p, 2q11.1-q35	4-6 (36.4-54.5)	0-2 (0-2.5)	<0.01
3p, 3q	4-7 (36.4-63.6)	1-3 (1.3-3.8)	<0.01
4p15.1-p16.3, 4q25-q26, 4q28.2-q35.1	4-6 (36.4-54.5)	4-6 (5.0-7.5)	<0.01
5q11-q13.2, 5p	5-7 (45.5-63.6)	5-7 (6.3-8.8)	<0.01
6p11.1-p12.1, 6p21.1-p25.3, 6q12-q27	3-5 (27.3-45.5)	1-3 (1.3-3.8)	<0.01
7p11.2-p22.3, 7q	4-5 (36.4-45.5)	0-4 (0-5.0)	<0.01
8p11.1-p12, 8q21.11-q21.12, 8q21.2	6-7 (54.5-63.6)	6-10 (7.5-12.5)	<0.01
9p13.2-p24.3, 9q	5-8 (45.5-72.7)	3-5 (3.8-6.3)	<0.01
10p11.22-p12.33, 10q21.1-q24.2	4-6 (36.4-54.5)	1-4 (1.3-5.0)	<0.01
10q24.32-q26.3	4-6 (36.4-54.5)	1-4 (1.3-5.0)	<0.01
11p, 11q11-q13.4, 11q14.1-q25	4-7 (36.4-63.6)	2-6 (2.5-7.5)	<0.01
12p, 12q	6-8 (54.5-72.7)	4-7 (5.0-8.8)	<0.01
13q21.2-q32.1, 13q33.1-33.3	8-9 (72.7-81.8)	18-19 (22.5)	<0.01
14q12-q13.2	4-5 (36.4-45.5)	3-4 (3.8-5.0)	<0.01
15q13.1-14, 15q21.3	3-5 (27.3-45.5)	1-4 (1.3-5.0)	<0.01
15q22.32-q22.33, 15q24.2-q26.3	3-5 (27.3-45.5)	1-4 (1.3-5.0)	<0.01
16p11.2-p13.3, 16q22.2-24.3	3-5 (27.3-45.5)	0-3 (0-3.8)	<0.01
19p12-p13.11, 19q13.12-q13.43	4-6 (36.4-54.5)	6-8 (7.5-10.0)	<0.01
21q11.2-q22.3	4-5 (36.4-45.5)	2-3 (2.5-3.8)	<0.01
22q11.1-q11.21, 22q11.23-13.33	4 (36.4)	1-2 (1.3-2.5)	<0.01
CNLOH			
15q22.1-q26.3	3-4 (27.3-36.4)	0-1 (0-1.3)	<0.01
22q11.1-q12.2, 22q13.1-q13.33	4 (36.4)	0-2 (0-2.5)	<0.01
17p11.2-p13.3	3 (27.3)	1-2 (1.3-2.5)	<0.01
10q23.22-q26.3	3 (27.3)	0	<0.01
1p22.1-p22.3, 1p34.2, 1p36.12-p36.23	2-3 (18.2-27.3)	0-1 (0-1.3)	<0.05
8p21.1-23.3	2 (18.2)	0	<0.05
LOH			
17p, 18p, 18q12.1, 18q12.3-q23	5-7 (45.5-63.6)	3-5 (3.8-6.3)	<0.01
14q23.1-q24.1, 14q31.1	4 (36.4)	2-3 (2.5-3.8)	<0.01
20p12.1	3 (27.3)	1 (1.3)	<0.01
6q14.2-q15	2 (18.2)	0	<0.05
14q22.1-q22.3, 14q24.2-q24.3, 14q31.2-31.3	3 (27.3)	2 (2.5)	<0.05
15q15.2-q21.2	3 (27.3)	2 (2.5)	<0.05
